# A Rare Presentation of Metastatic Extragonadal Nonseminomatous Germ Cell Tumor Complicated by Suspected Transfusion-Associated Circulatory Overload

**DOI:** 10.7759/cureus.113535

**Published:** 2026-07-28

**Authors:** Nefia Chacko, Rahul Roshin, Taner B Celebi

**Affiliations:** 1 Family Medicine, New York Institute of Technology, Old Westbury, USA; 2 Family Medicine, Donald and Barbara Zucker School of Medicine at Hofstra/Northwell, Plainview, USA; 3 Family Medicine, Northwell Health, Commack, USA

**Keywords:** anemia, extragonadal germ cell tumor, metastatic disease, nonseminomatous germ cell tumor, transfusion-associated circulatory overload (taco), transfusion-related acute lung injury (trali), transfusion safety

## Abstract

Extragonadal nonseminomatous germ cell tumors (NSGCTs) are rare and aggressive malignancies that commonly present with advanced metastatic disease and elevated tumor markers. We present the case of a 27-year-old man with no significant past medical history who presented with abdominal pain, dark stools, fatigue, weight loss, and severe normocytic anemia. Imaging revealed a large retroperitoneal mass with extensive pulmonary and hepatic metastases, while tumor marker evaluation demonstrated markedly elevated beta-human chorionic gonadotropin levels without evidence of a primary testicular lesion, consistent with metastatic extragonadal NSGCT. The patient required multiple packed red blood cell (pRBC) transfusions for progressive anemia, and subsequently developed acute hypoxic respiratory failure with pulmonary edema concerning for transfusion-associated circulatory overload (TACO). Despite intensive care management, embolization for active hepatic bleeding, and initiation of chemotherapy, the patient experienced progressive clinical deterioration and expired following cardiac arrest. This case highlights the aggressive clinical course of metastatic extragonadal NSGCT and the challenges associated with transfusion-related pulmonary complications in critically ill oncology patients. It also emphasizes the importance of careful transfusion practices, clinical vigilance, and early recognition of transfusion reactions in patients with significant inflammatory burden, ongoing hemorrhage, and extensive metastatic disease.

## Introduction

Testicular germ cell tumors (GCTs) make up most testicular malignancies and are the most common solid tumors in young adult males [1,2]. GCTs are broadly categorized histologically into seminomas and nonseminomatous germ cell tumors (NSGCTs); seminomas tend to be more homogeneous and radiotherapy-sensitive, whereas NSGCTs are heterogeneous and include embryonal carcinoma, yolk sac tumor, choriocarcinoma, and teratoma, and are typically more aggressive and more likely to metastasize [3,4]. NSGCTs characterize a substantial proportion of testicular GCTs. They are associated with elevated tumor markers, such as alpha-fetoprotein (AFP), beta-human chorionic gonadotropin (β-hCG), and lactate dehydrogenase (LDH), which are used in diagnosis, staging, and prognostication [2].

GCTs mainly present as a painless testicular mass, whereas a significant subset of patients with nonseminomatous tumors may initially present with symptoms attributable to metastatic disease, including gastrointestinal, pulmonary, or constitutional symptoms [1,4]. Although GCTs most commonly arise within the testes, a small but clinically important subset, approximately 5%, occurs in extragonadal sites without detectable testicular primary tumors, most frequently along midline structures such as the retroperitoneum and mediastinum [5]. Extragonadal GCTs present with symptoms of mass effect and advanced disease, and their management is similar to that of metastatic testicular GCTs, though prognosis can vary by histological subtype and location [5]. These disease characteristics have important implications for clinical care, as NSGCTs are more aggressive in nature. Patients with advanced disease often develop severe anemia from their underlying malignancy or tumor-associated bleeding, requiring blood transfusions as part of supportive management. While transfusion is frequently necessary, it also places these patients at risk for serious transfusion-related pulmonary complications.

Transfusion-associated circulatory overload (TACO) and transfusion-related acute lung injury (TRALI), in particular, are the most common pulmonary complications following blood product transfusion and may present with similar clinical features, including acute respiratory distress and pulmonary infiltrates within six hours of transfusion [6,7]. However, these reactions have different pathophysiology and management, making accurate diagnosis important. Prompt distinction between TACO and TRALI is essential because their management strategies differ significantly. TRALI is characterized by a non-cardiogenic pulmonary process driven by inflammatory mechanisms that increase pulmonary capillary permeability due to immune activation or donor-derived mediators [7,8]. In contrast, TACO results from hydrostatic pulmonary edema due to impaired tolerance of intravascular volume expansion and elevated pulmonary capillary pressures. Current research supports a two-hit model for TACO, in which systemic inflammation, malignancy, end-organ dysfunction, or reduced physiologic reserve predisposes patients to pulmonary edema, with transfusion serving as the precipitating insult [9].

In this case report, we describe a young man with metastatic extragonadal NSGCT who initially presented with gastrointestinal symptoms and severe anemia requiring red blood cell transfusion. His clinical course was subsequently complicated by suspected TACO, highlighting the diagnostic challenge of distinguishing TACO from TRALI and other causes of acute respiratory failure in patients with advanced malignancy. This case further emphasizes that TACO may occur even in younger patients without traditional cardiovascular risk factors, likely due to the physiologic compromise associated with advanced metastatic disease.

## Case presentation

A 27-year-old man with no significant past medical history presented to the emergency department (ED) with diarrhea, abdominal pain, and dark stools. He reported feeling unwell for approximately two weeks, with symptoms including progressive nausea, vomiting, fatigue, decreased exercise tolerance, and an unintentional 10-lb weight loss. His illness initially began with constipation, for which he self-administered laxatives for several days. He subsequently developed loose, black stools, prompting discontinuation of laxative use approximately four days prior to presentation, though the black stools persisted. The patient also endorsed upper abdominal pain, dizziness upon standing, and feeling dehydrated, with pallor noted by others. He was able to tolerate liquids and fruit but reported emesis with most solid foods. He denied fever but reported intermittent chills. At symptom onset, he had taken bismuth subsalicylate.

On presentation, vital signs were notable for blood pressure 109/62 mmHg, heart rate 92 beats/min, respiratory rate 17 breaths/min, temperature 36.3 °C, and oxygen saturation 97% on room air. Physical examination revealed pallor and mild epigastric and left upper quadrant tenderness without peritoneal signs.

Laboratory evaluation demonstrated normocytic anemia with hemoglobin 6.4 g/dL, hematocrit 19.8%, and mean corpuscular volume 81.1 fL. Iron studies showed low serum iron, unsaturated iron-binding capacity, total iron-binding capacity, and transferrin, with markedly elevated ferritin levels. LDH and haptoglobin were elevated, while direct antiglobulin (Coombs) testing and fecal occult blood testing were negative. Although the negative fecal occult blood test may have reflected bismuth-related stool discoloration, the patient reported black stools preceding bismuth therapy. Overall, these lab findings were most consistent with anemia of chronic disease, as summarized in Table 1. However, given the acutely low hemoglobin requiring transfusions and suspicion for a source of bleed, this may suggest a multifactorial etiology to the patient’s anemia.

**Table 1:** Patient's laboratory values on initial presentation, with reference ranges for all values, respectively.

Lab	Patient’s value	Reference range
Hemoglobin	6.4	13.0-17.0 g/dL
Hematocrit	19.8%	39.0%-50.0%
Mean corpuscular volume (MCV)	81.1	80.0-100.0 fL
Serum iron, total	17	45-165 ug/dL
Unsaturated iron-binding capacity (UIBC)	150	110-370 ug/dL
Total iron-binding capacity (TIBC)	166	220-430 ug/dL
% iron saturation	19%	16%-55%
Ferritin	858	30-400 ng/mL
Serum transferrin	140	200-360 mg/dL
Serum lactate dehydrogenase (LDH)	293	135-225 u/L
Serum haptoglobin	297	34-200 mg/dL
ABO Rh interpretation	A+	-
Direct antiglobulin (Coombs)	Negative	-
Fecal occult blood testing (FOBT)	Negative	-

**Figure 1:**
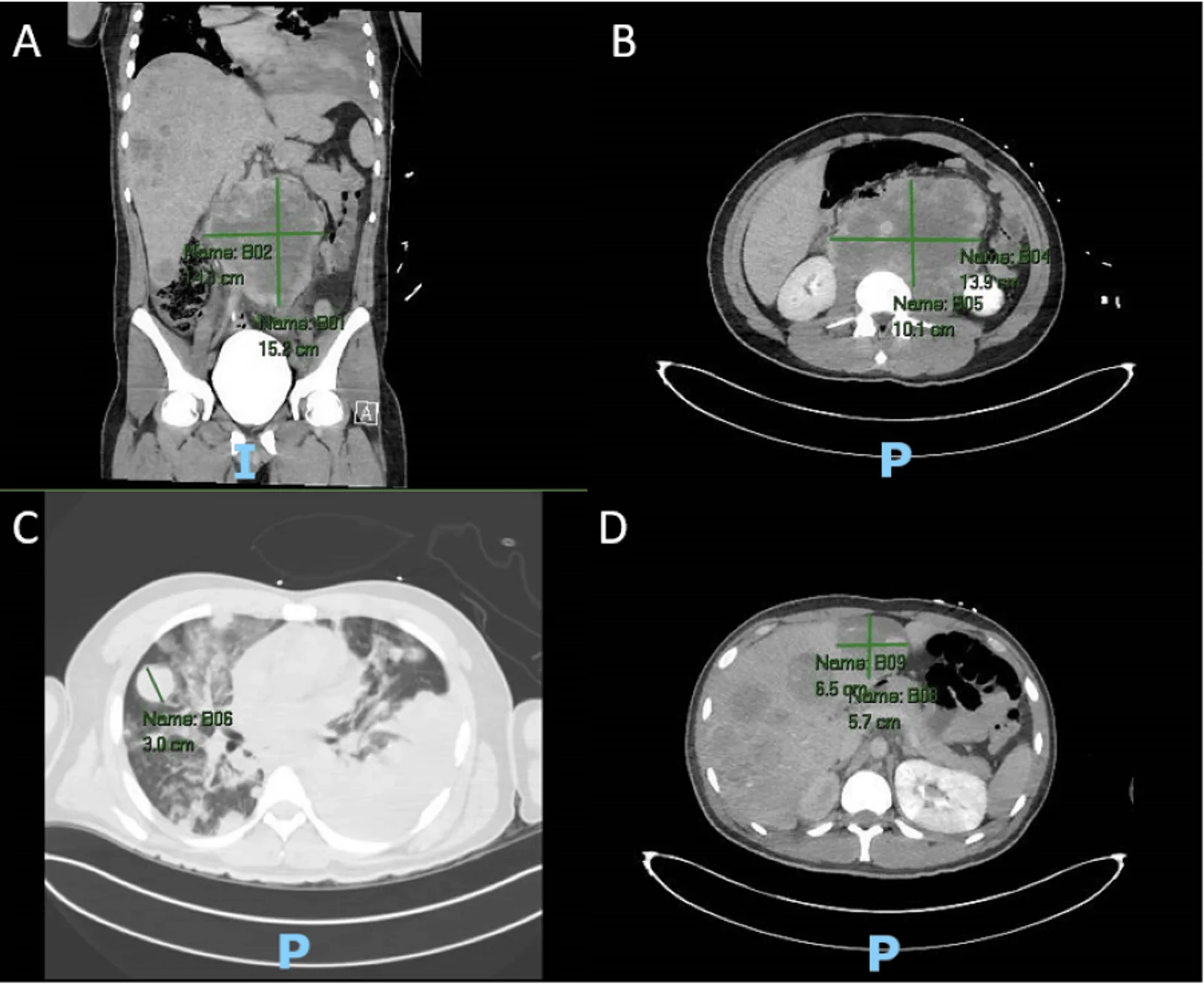
CT of the abdomen and pelvis with intravenous contrast. Contrast-enhanced computed tomography of the abdomen and pelvis, in coronal (A) and transverse (B) views, demonstrating a large retroperitoneal mass measuring approximately 14.3 cm x 15.2 cm and 10.1 cm x 13.9 cm, respectively, encasing the abdominal aorta (beginning just below the level of the superior mesenteric artery), bilateral renal arteries, inferior mesenteric artery, and left common iliac artery. The mass abuts and extrinsically compresses the inferior vena cava, left common iliac vein, and bilateral psoas muscles (left greater than right). Imaging also demonstrates numerous bilateral pulmonary nodules, at least 25, measuring up to 3 cm in size (C) and numerous hypoattenuating hepatic lesions, at least 20, with the largest being 6.5 cm x 5.7 cm (D).

Computed tomography of the abdomen and pelvis demonstrated a large retroperitoneal mass encasing the abdominal aorta (below the level of the superior mesenteric artery), bilateral renal arteries, inferior mesenteric artery, and left common iliac artery, with extrinsic compression of the inferior vena cava and left common iliac vein, as well as numerous pulmonary nodules and multiple hepatic lesions, consistent with widely metastatic malignancy. Associated mild left hydroureteronephrosis was also noted, as seen in Figure 1.

He received one unit of packed red blood cells (pRBCs) with hemoglobin improvement to 7.6 g/dL. Hematology/oncology consultation prompted further evaluation, including tissue biopsy, chest imaging, echocardiography, testicular ultrasound, and tumor marker assessment. Tumor markers demonstrated markedly elevated β-hCG (458,746 mIU/mL) and LDH (905 U/L), with no testicular mass identified on ultrasound. Computed tomography of the chest revealed a small right lower-lobe segmental pulmonary embolism, while transthoracic echocardiography showed preserved biventricular function with a left ventricular ejection fraction of 62%. The patient was initiated on intravenous heparin and transitioned to apixaban after close discussion with hematology/oncology, given no definite source for an acute bleed upon inpatient workup and clinical improvement in the patient’s symptoms at that time. This was done with understanding of the risks and benefits of the imaging findings, given no true zero-risk option for management of the patient’s pulmonary embolism. On the day prior to discharge, hemoglobin decreased to 7.0 g/dL, prompting transfusion of a second unit of pRBCs. After stabilization of hemoglobin levels with no observed signs of acute bleeding, the patient was discharged with a plan for close outpatient follow-up with hematology/oncology regarding pathology results and monitoring of anemia, as well as with gastroenterology for additional monitoring of anemia.

Two days later, the patient was brought via ambulance to an affiliated hospital with chest pain. He denied dyspnea, cough, prior similar episodes, abdominal pain, back pain, or overt evidence of bleeding but reported persistent nausea and poor oral intake since hospital discharge. On arrival, vital signs were notable for blood pressure 107/59 mmHg, heart rate 117 beats/min, respiratory rate 30 breaths/min, temperature 38.1 °C, and oxygen saturation 97% on 6 L/min nasal cannula. Laboratory evaluation revealed anemia with a hemoglobin of 5.3 g/dL and hematocrit of 15.8%.

The patient was admitted to the medicine service again for management of anemia secondary to metastatic malignancy with concurrent evaluation for a possible source of bleeding. While awaiting inpatient bed placement in the emergency department, the patient received one unit of pRBCs, over the course of an hour, with initiation of a second unit shortly thereafter.

**Figure 2:**
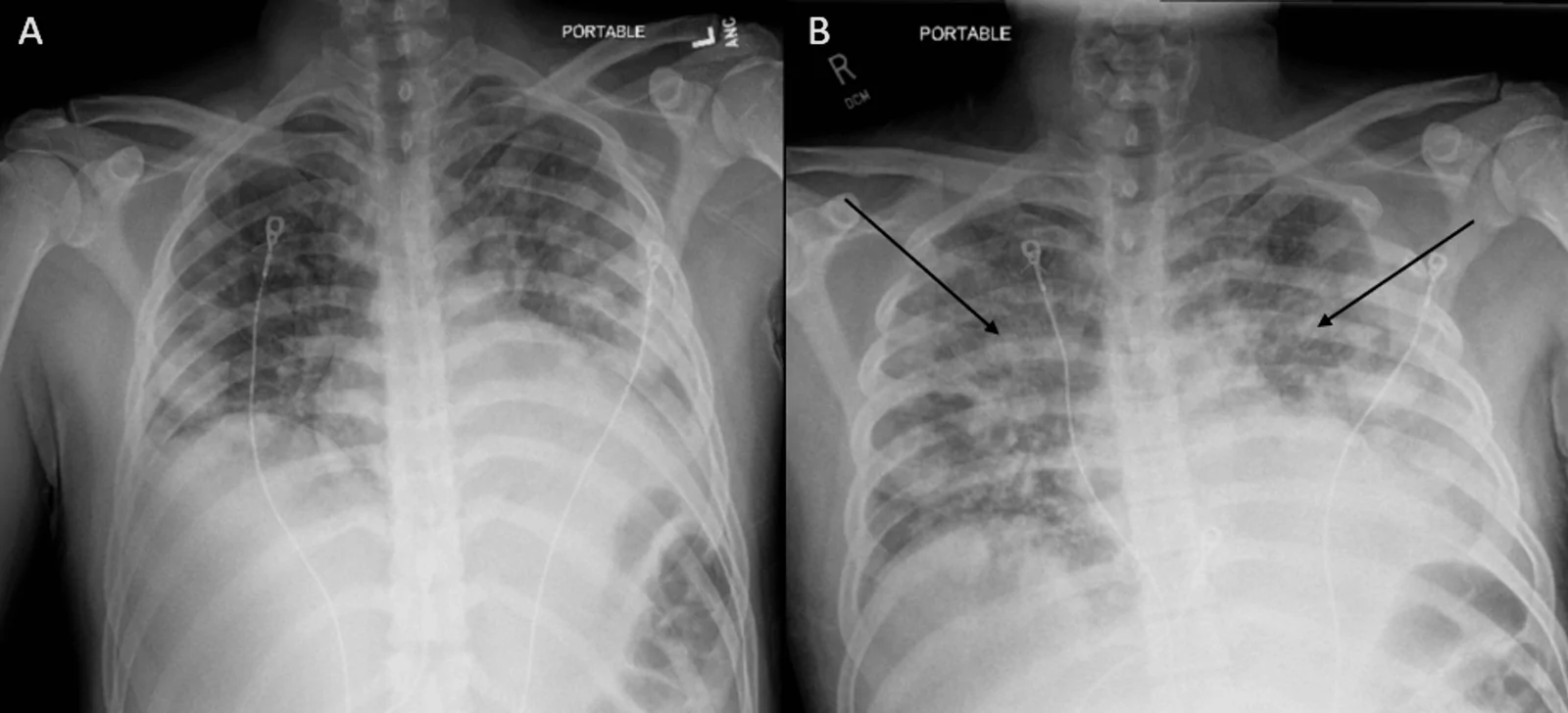
Chest X-ray before and after transfusion. Chest X-ray, pre-symptom onset, illustrating diffuse bilateral pulmonary opacities suspicious for metastatic disease versus less likely pneumonia (A). Chest X-ray, post-symptom onset, re-demonstrating innumerable bilateral lung nodules and now increasing airspace opacities suggestive of superimposed pneumonia or pulmonary edema with bilateral pleural effusions (black arrows) (B).

Approximately two hours after completion of the first unit of pRBCs, the patient developed profuse diaphoresis and increased work of breathing. Physical examination revealed bilateral pulmonary crackles. Vital signs were notable for tachypnea (respiratory rate 30-40 breaths/min), tachycardia (heart rate 133 beats/min), low-grade fever (38.8 °C), blood pressure 126/65 mmHg, and oxygen saturation 94%-96% on a non-rebreather mask. Given concern for a transfusion reaction, the second unit of pRBCs was immediately discontinued. Urgent evaluation, including stat laboratory studies and chest radiography, demonstrated findings consistent with acute pulmonary edema and volume overload, while hemolytic transfusion reaction and disseminated intravascular coagulation evaluations were negative. With initiation of diuretic therapy (in this case, intravenous bumetanide) and discontinuation of the second unit of blood, as well as supplemental oxygen and opioids for analgesic therapy, the patient slowly began to report improvement in his symptoms. The results of the aforementioned hematologic workup are detailed in Table 2, with corresponding chest X-ray result in Figure 2.

**Table 2:** Patient’s laboratory values following suspected transfusion reaction. Samples were collected while the patient was on 100% non-rebreather.

Lab	Patient’s value	Reference range
Hematology		
Hemoglobin	4.9	13.0-17.0 g/dL
Hematocrit	14.9%	39.0%-50.0%
Platelets	271	150-400 K/uL
Renal studies		
Blood urea nitrogen (BUN)	18	7-23 mg/dL
Creatinine	1.42	0.50-1.3 mg/dL
Estimated glomerular filtration rate (eGFR)	69	>60 mL/min/1.73 m^2^)
Hemolysis markers		
Total bilirubin	2.5	0.2-1.2 mg/dL
Indirect reacting bilirubin	0.9	0.2-0.8 mg/dL
Serum haptoglobin	40	34-200 mg/dL
Transfusion-related studies		
ABO Rh interpretation	A+	-
Direct antiglobulin (Coombs)	Negative	-
Other		
Pro-brain natriuretic peptide (pBNP)	183	0-300 pg/dL
Arterial blood gas*		
pH	7.36	7.35-7.45
pCO2	35	35-45 mmHg
pO2	92	80-100 mmHg
HCO3	20	22-26 mEq/L

The patient was transferred to the ICU for escalating respiratory failure. IR was consulted for suspected ongoing hemorrhage, and triple-phase computed tomography angiography of the abdomen and pelvis revealed bilobar hepatic lesions with contrast enhancement concerning for active bleeding. Hepatic angiography followed by gelfoam embolization of the right hepatic artery was performed. Post-procedure, the patient required additional transfusion support, receiving a total of three units of pRBCs before transfer to another ICU for initiation of inpatient chemotherapy. Due to worsening respiratory failure requiring high-flow nasal cannula, a VIP chemotherapy regimen was selected over BEP per hematology/oncology recommendations. This decision was made due to the presence of extensive lung metastases to limit the risk of bleomycin-associated pulmonary toxicity, given comparable results in treatment of GCTs when compared to BEP [10].

Following transfer, surgical pathology results became available, demonstrating findings overall consistent with an underlying NSGCT. Samples were obtained of the retroperitoneal mass via a left anterior abdominal approach by interventional radiology.

Sections showed predominantly necrotic tissue admixed with hemorrhage and scattered malignant-appearing cells with poor preservation. Occasional multinucleate cells suggestive of syncytiotrophoblasts were present. Immunohistochemical stains performed showed scattered cells positive for OCT3/4, with rare cells labeling for inhibin. GATA3 highlighted scattered multinucleate cells, with cytokeratin AE1/AE3 highlighting neoplastic cells. CD30, PLAP, and p63 markers were noted to be negative.

Additional immunostaining performed at an outside laboratory to further classify the neoplasm showed neoplastic cells positive for HCG, but negative for SALL4. Per pathology reporting, while the HCG positivity was indicative of trophoblastic differentiation and may represent a choriocarcinoma or other GCT with syncytiotrophoblasts, further histologic classification was limited due to extensive necrosis, poor tumor viability, and a lack of lesional cells.

Despite initiation of VIP chemotherapy, the patient experienced progressive hypoxic respiratory failure, necessitating endotracheal intubation, followed by hemodynamic instability requiring vasopressor support. On the following day, he suffered cardiac arrest in the setting of concern for ongoing hemorrhage. Massive transfusion protocol and advanced cardiac life support were initiated; however, resuscitative efforts were discontinued per family wishes, and the patient was pronounced deceased.

## Discussion

TACO is the leading cause of transfusion-related morbidity and mortality, with an estimated incidence of approximately 2.2 cases per 1,000 transfused units, whereas TRALI is the second leading cause of transfusion-related mortality and is considerably less common, with reported incidences ranging from 0.02 to 0.10 per 10,000 transfused blood components [11-14]. TACO most commonly affects older adults and patients with cardiac or renal dysfunction, while TRALI is associated with acute critical illness, liver or kidney dysfunction, shock, perioperative transfusion, and positive fluid balance [7].

According to the criteria set by the CDC National Healthcare Safety Network, TACO is defined as new onset or exacerbation of three or more of the following within 12 hours of cessation of a transfusion: (1) acute or worsening respiratory compromise (dyspnea, tachypnea, hypoxemia); (2) acute or worsening pulmonary edema, either clinical (crackles, orthopnea, pink frothy sputum, S3) or radiographic; (3) cardiovascular changes not explained by the underlying condition; (4) evidence of fluid overload; and (5) elevated BNP or NT-proBNP above the reference range or a significant rise from baseline [15].

In this case, the patient received 1one unit of pRBCs over one hour and developed acute respiratory distress approximately two hours after completion of the transfusion. He became hypoxemic, with an oxygen saturation of 80% on room air, leading to escalation to a non-rebreather mask, which improved his oxygen saturation to the mid-90% range. He was also markedly tachypneic, with a respiratory rate of 30-40 breaths/min, and reported dyspnea. Additionally, he was tachycardic, with a heart rate in the 130s. On physical examination, crackles were noted on lung auscultation. A post-transfusion chest radiograph demonstrated new airspace opacities concerning for pulmonary edema with bilateral pleural effusions, findings that were absent on pre-transfusion imaging. The patient also exhibited cardiovascular changes, including tachycardia and a widening of pulse pressure (126/65 mmHg; pulse pressure 61 mmHg). Collectively, these findings supported TACO as a likely etiology of the patient's post-transfusion respiratory deterioration, given the presence of acute respiratory compromise, new pulmonary edema, and cardiovascular changes consistent with circulatory overload.

Although the patient was young, multiple risk factors, including advanced metastatic malignancy, anemia, active bleeding, systemic inflammation, and repeated transfusions, may decrease tolerance to intravascular volume expansion. This case emphasizes the need for heightened awareness of TACO even in patients without cardiac risk factors and underscores the importance of careful transfusion practices, and early recognition of transfusion-related pulmonary complications.

## Conclusions

This case illustrates the importance of maintaining a high index of suspicion for transfusion-associated pulmonary complications in patients with advanced cancer receiving blood transfusions. This patient developed acute respiratory deterioration with new pulmonary edema shortly after transfusion, raising concern for TACO while highlighting the importance of carefully distinguishing TACO from TRALI and other potential causes of respiratory failure. In patients with advanced malignancy, extensive metastatic disease, and severe anemia requiring transfusions, clinical evaluation using established diagnostic criteria, including assessment for respiratory compromise, pulmonary edema, cardiovascular changes, fluid status, and supportive biomarkers, is required to guide management. Cautious transfusion practices, including pre-transfusion risk assessment in susceptible patients, and early recognition of transfusion-related adverse reactions are critical to minimizing patient morbidity and facilitating timely intervention. Overall, this case underscores both the aggressive clinical course of metastatic NSGCT and the need for vigilant monitoring and individualized transfusion practices in critically ill oncology patients.
